# Fish Bone Causing Perforation of the Intestine and Meckel's Diverticulum

**DOI:** 10.1155/2020/8887603

**Published:** 2020-09-19

**Authors:** Fakhar Shahid, Samir Omer Abdalla, Tamer Elbakary, Ahmad Elfaki, Syed Muhammad Ali

**Affiliations:** ^1^Department of Surgery, Hamad Medical Corporation, Doha, Qatar; ^2^Department of Surgery, Al-Wakra Hospital, HMC, Al-Wakra, Qatar; ^3^Department of Acute Care Surgery, Hamad Medical Corporation, Doha, Qatar

## Abstract

Perforation of small bowel due to ingested fish bone is rare, the most common site is ileum and occasionally, it can involve the appendix and/or Meckel diverticulum. We report six patients who, developed bowel perforation after fish bone ingestion, four of them found to have rent in the ileum and two through Meckel's diverticulum and presented with abdominal pain and localized peritonitis. All underwent surgical exploration and removal of the fish bone and closure of the small intestine/excision of the diverticulum. Foreign body ingestion should be kept in mind in suspicious cases, and laparoscopy is very important to diagnose such rare cases as they may commonly be missed by imaging.

## 1. Introduction

Perforation of the gastrointestinal (GI) tract due to an ingested fish bone (FB) is a rare event occurring in less than 1 percent of patients [1, 2]. It is generally known that most pass uninterrupted through the gastrointestinal tract without causing any symptoms or perforation [3, 4]. Injury may occur anywhere from mouth to anus [5]. However, rarely, perforation has also been described in Meckel's diverticulum and in the appendix [6]. Preoperative diagnosis is usually difficult, and the dietary history is not very helpful because fish bone ingestion is common and easily forgotten [7]. We report six cases with acute abdomen, who were found to have fish bone perforating the small bowel and their operative management.

## 2. Method

We collected the data of six patients from the Cerner record system of Hamad General Hospital, Doha-Qatar, over the period of 3 years, i.e., from 2016 to 2019. The study involved all the patients with intraoperative fish bone perforating the small bowel. The record included the patient's demographics, presenting symptoms, imaging, and intraoperative findings.

## 3. Results

All patients had history of pain of 1-4 days duration. All of them had localized right lower quadrant abdominal pain mimicking acute appendicitis. None of the patients reported nausea, vomiting, anorexia, or fever. There was no history of bleeding per rectum or constipation. All of our patients were healthy and young with no past medical or family history. The age ranged from 24-48 (mean 37 yrs.).  Table 1 summarizes all the details of six cases.

On examination, the patients were hemodynamically stable without signs of sepsis, tachycardia, or hypotension. The abdominal examination revealed tenderness in the right lower quadrant along with rebound in all of the cases. There was no palpable masses. All of them had high white blood cell counts in the range of 9 × 10^9^/l to 15 × 10^9^/l and C-reactive protein in the range of 12 to 98 mg except case number 3 who had normal white cell count but high C-reactive protein. All the patients underwent a computed tomography (CT) scan of abdomen with oral and intravenous contrast except cases number five and six.

**Figure 1:**
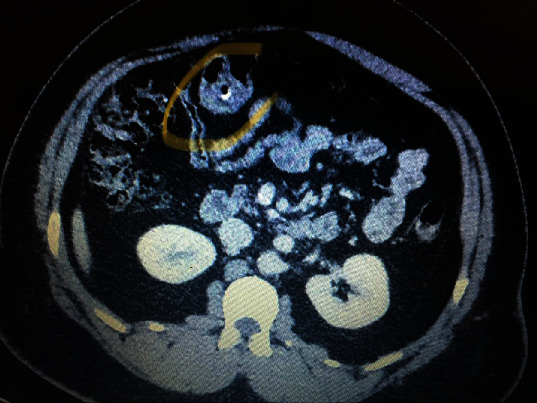
CT scan of case number 1.

**Figure 2:**
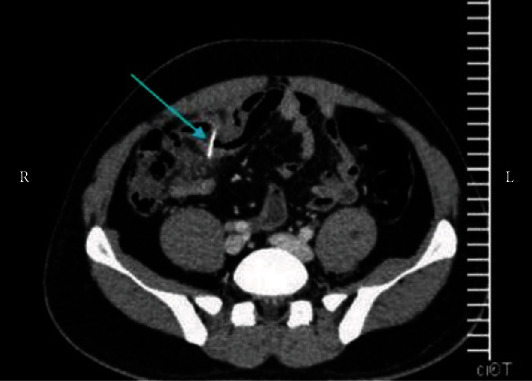
CT scan of case number 2 showing foreign body in the ileum (arrow).

**Figure 3:**
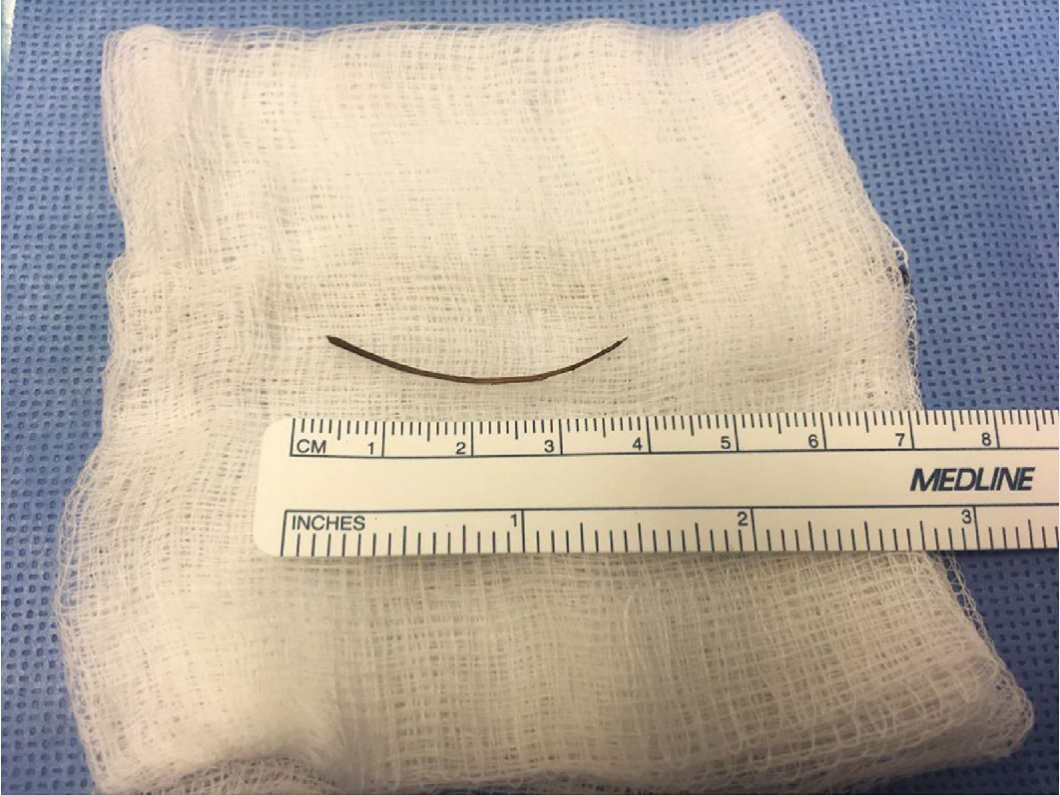
Postoperative fish bone specimen in case number 4.

The CT scan of case number 1 showed normal appendix and some mesenteric lymph nodes but no foreign body. The patient had continuous right abdominal pain which was not relieved with analgesia, so he was taken to the operating room for diagnostic laparoscopy. A fish bone was seen penetrating the wall of the ileum. It was safely dissected out, and the defect was primarily repaired with 3/0 PDS. Retrospectively, the CT scan was discussed with the radiologist, and the foreign body was pointed out to be seen in the image as described in Figure 1. In the cases 2 and 4, the CT scan reported normal appendix and a foreign body in the ileum perforating the bowel. Diagnostic laparoscopy was performed, and the foreign body was retrieved. In patient 4, there was some purulent fluid in the pelvis and right iliac fossa that was sucked out. The perforation was sutured with PDS suture. (Figures 2 and 3).

**Figure 4:**
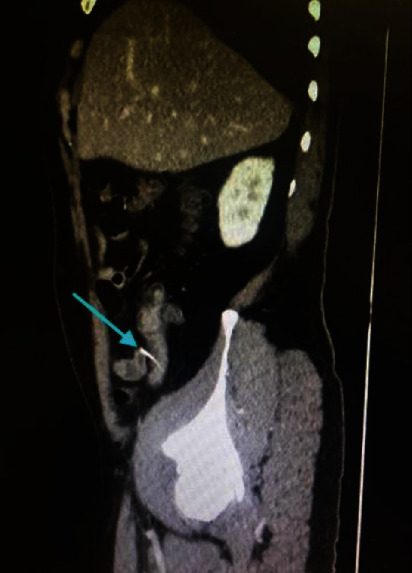
CT scan of case number 3 showing foreign body (arrow).

**Figure 5:**
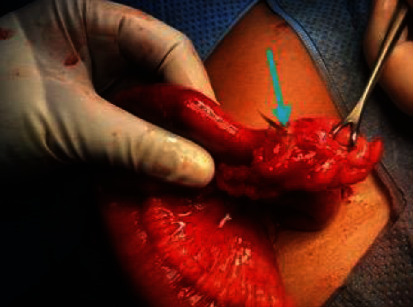
Foreign body seen perforating Meckel's diverticulum in case 5 (arrow).

In case number 3, the CT scan did not show foreign body but it showed inflammatory changes in the appendix and adjacent cecal wall. He underwent diagnostic laparoscopy with foreign body removal from the terminal ileum and appendectomy. The perforation was sutured. The histopathology showed normal appendix. Similar to the first case, the foreign body was not reported either, before the CT scan. (Figures 4 and 5).

**Figure 6:**
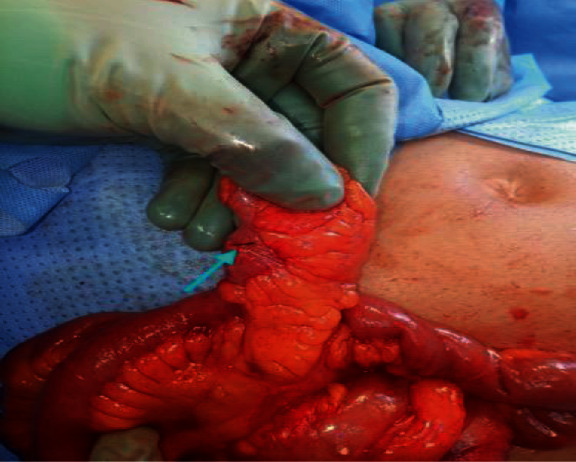
Meckel's diverticulum containing the fish bone penetrating it in case 6.

**Figure 7:**
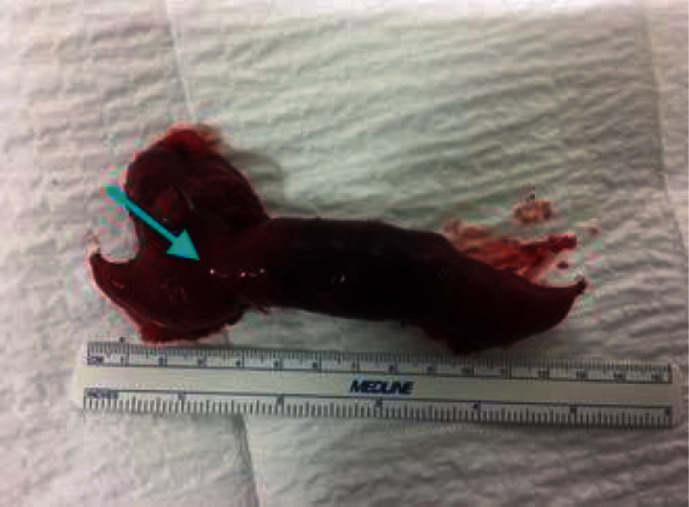
Meckel's diverticulum specimen case 5.

In cases 5 and 6, the clinical picture and abdominal examination was consistent with acute appendicitis. An open procedure by grid iron incision was carried out as the patients were thin and lean. We found the fish bone perforating Meckel's diverticulum in both cases. The fish bone was removed along with the resection of the segment of small bowel with perforated Meckel's diverticulum as the base of both diverticulae were thick and had abnormal consistency especially the case number 6 (Figure 6). The continuity of the bowel was restored by side-to-side anastomosis using GIA (gastrointestinal anastomosis) stapler along with appendectomy (Figures 6 and 7). Histopathology of Meckel's diverticulum, however, did not show any abnormal mucosa, whereas that of appendix revealed mild inflammation in both. All patients tolerated the surgical procedures well and had unremarkable postoperative course and were discharged within 3 to 5 days after the surgery. All patient received a single dose of intravenous cefuroxime 1.5 grams at the induction of anesthesia, and no postoperative antibiotics were prescribed as the peritoneal cavity was mopped dry. Follow-up visits at two weeks and three months did not show any complaints or complications.

## 4. Discussion

Fish bones, chicken bones, and tooth picks are the most common foreign materials to cause bowel perforation due to their sharp ends [2, 8]. However, pens, nails, nail clippers, batteries, and pegs are also reported to cause intestinal perforation [9]. Goh et al. reported that swallowed fish bones are the most common cause of gastrointestinal perforation because of their sharp tips and long bodies [10]. In our cases, we had six patients who were found to have fish bone perforation in the ileum including two cases with perforated Meckel's diverticulum. Fish bones usually perforate the sites with acute angulations such as the ileocecal junction, ileum, or the flexures of the colon [3, 7, 10] and rarely involve Meckel's diverticulum or appendix [11]. Patients usually present with acute acute abdominal pain l but the diagnosis is often missed as this is a rare incident, and they usually do not recall fish bone ingestion [12]. In our cases, none of the patients were asked about the history of fish ingestion preoperatively, and only half of them gave the history of fish intake couple of days prior to the abdominal pain. Some of the patients present with pain at the lower abdomen especially in the right side mimicking acute appendicitis as was the case with two of our patients. Different signs have been described including localized abdominal abscess, colorectal, colovesical, and enterovesical fistula, inflammatory mass, chronic or acute intestinal obstruction, bleeding, endocarditis, renal, and ureteric colic [11, 13, 14]. But very often, these patients can be asymptomatic as well [15]. It is not easy to detect fish bones in the CT scan, and it was done in four of our cases of acute abdomen and was not picked up by the radiologist in two [16]. The presence of pneumoperitoneum is not reliable as it is not found in many of cases [13]. This is because the perforation is usually caused by the impaction and progressive erosion of the FB through the intestinal wall, allowing it to be covered by fibrin, omentum, or adjacent loops of bowel. This limits the passage of large amounts of intraluminal air into the peritoneal cavity [4].

Management of these patients can fall under two categories: either to perform laparoscopic exploration as we carried out in four of our patients or take them for open surgery by laparotomy or traditional appendectomy incision as we did in two patients who were found to have perforation of Meckel's diverticulum despite that they had all the classic clinical features of acute appendicitis and therefore, no imaging was done. Most of the previously reported cases were managed operatively with the resection of small bowel and anastomosis [3, 17]. The benefit of laparoscopy for appendicectomy and as a tool for the initial exploration of abdominal sepsis has helped in diagnosing this type of rare condition, avoiding the need of laparotomy in many patients [18].

## 5. Conclusion

This case series stresses the importance of excluding other diagnosis such as foreign body perforation in individuals with abdominal pain. Despite the availability of imaging, unexpected diagnoses can still be missed and are usually made intraoperatively. Early diagnostic laparoscopy plays an important role as it helps intraoperatively to diagnose as well as to decide what surgical corrective intervention is required.

## Figures and Tables

**Table 1:** Summarizes the presentation of all the cases.

Case no.	1	2	3	4	5	6
Sex and age	29 M	32 M	48 M	24 M	45 M	42 M
Clinical features						
Pain duration	1 day	3 days	1 day	3 days	4 days	2 days
Localization of pain	1	1	1	1	1	1
CRP	20	98	143	27	Not done	12
WBC	15000	12000	9000	11000	12000	14000
CT findings						CT not done
Abscess	0	0	0	0	0	0
Thickened ileal loop	0	1	1	1	0	0
Fatty infiltration	0	0	1	1	1	0
Localized pneumoperitoneum	0	0	0	0	0	0
Peritoneal effusion	0	0	0	1	0	0
Calcified foreign body	1^∗^	1	0	1	0	0
Treatment	DL	DL	DL+LA	DL	OA	OA
Site of perforation (intraoperative)	Ileum	Ileum	Ileum	Ileum	Meckel's	Meckel's
Bowel resection	No	No	No	No	Yes	Yes

DL: diagnostic laparoscopy; AO: open appendectomy; LA: laparoscopic appendectomy; ^∗^retrospective; Yes: 1, No: 0.
